# *miR-155* inhibitor reduces the proliferation and migration in osteosarcoma MG-63 cells

**DOI:** 10.3892/etm.2014.1942

**Published:** 2014-09-01

**Authors:** HUICHENG LV, JUN GUO, SIQIN LI, DIANMIN JIANG

**Affiliations:** 1 Department of Orthopaedics, The First Affiliated Hospital of Chongqing Medical University, Chongqing 404000, P.R. China; 2 Department of Orthopaedics, The Second Affiliated Hospital of Inner Mongolia Medical University, Hohhot, Inner Mongolia 010030, P.R. China; 3 Department of Ultrasound, The People’s Hospital of Inner Mongolia, Hohhot, Inner Mongolia 010010, P.R. China

**Keywords:** *miR-155* inhibitor, osteosarcoma, proliferation, migration

## Abstract

As the most common malignant primary bone tumor in childhood, osteosarcoma (OS) maintains a high recurrence, despite the significant improvements in the overall survival rate of high-grade OS patients during the recent decades. Therefore, a novel therapy strategy is required for OS treatment. Recently, various microRNAs (miRNAs or miRs) have been confirmed as deregulated in OS, and the *miR-155* dysregulation in OS has been discovered by the microarray analysis. In the present study, the regulation of *miR-155* on the OS cell proliferation, migration and invasion on the MG-63 cells was explored *in vitro*. The *miR-155* mimics were found to promote cell proliferation, colony formation, migration and invasion significantly, compared to the control miRNA. An *miR-155* inhibitor was also used to evaluate whether *miR-155* served as a therapeutic target for OS. The results demonstrated that the *miR-155* inhibitor significantly reduced the proliferation, colony formation, migration and invasion of the MG-63 OS cells. Thus, the study confirmed the oncogenic regulation on the OS progression of *miR-155*, which could serve as a therapeutic target with an *miR-155* inhibitor.

## Introduction

Osteosarcoma (OS) accounts for ~2.5% of all malignancies in pediatric patients and ~ 20% of all primary bone cancers (1), with a morphological and malignant heterogeneity (2). The majority of OS variant cells are extremely aggressive, with a capability of rapid growth and early metastasis. Currently, >30% of OS patients with localized disease eventually develop distant metastases, mostly to the lungs and bones (3), even following chemotherapy and surgical treatment. The outcome of OS patients has not significantly improved over the last 20 years, and there has been no significant advance in OS treatment, as the molecular mechanism underlying the highly efficient proliferation and migration of OS cells remains largely unknown. Thus, there is an urgency to identify the details regarding tumor progression and to develop novel therapy strategies for this disease.

microRNAs (miRNAs or miRs) are endogenous non-coding RNAs with 18–24 nucleotides, which regulate gene expression (4) by binding the target mRNA’s 3′ untranslated region (5), in a wide range of organisms, and in a broad array of cell processes in mammals (5–7). It is well known that cancer is driven by the deregulation of a complexity of oncogenic and tumor suppressive genes, and emerging evidence shows that miRNAs are deregulated in various types of cancer (8–10), and play oncogenic and tumor suppressive roles, contributing to tumor formation and development (11–13). Recently, various miRNAs have been confirmed to be deregulated in OS (14,15). The oncogenic miRNA, *miR-21*, which is aberrantly overexpressed in numerous types of tumor and induces cancer cell growth, migration, invasion and metastasis (16,17), has also been indicated to be significantly overexpressed in OS tissues and induces invasion and migration of the OS cell line, MG-63, by negatively regulating the tumor suppressor gene, reversion-inducing-cysteine-rich protein with kazal motifs (18). The oncogenic *miR-93* also induces proliferation and invasion in OS (19), whereas *miR-20a* promotes OS metastasis by regulating *Fas* expression (20). By contrast, the tumor suppressive miRNAs, including *miR-199a-3p* (21), *miR-125b* (22), *miR-143* (23), *miR-382* and *miR-134* (24), are significantly downregulated in OS cells and attenuate proliferation and inhibition of migration, reduce cell viability and induce apoptosis. *miR-155* is well identified as an oncogenic miRNA in leukemia (25,26) and breast cancer (14), contributing to tumorigenicity and progression.

Neoadjuvant chemotherapy has improved the cure rate of OS patients (27,28). However, patients that are not sensitive to these drugs have a poor prognosis. In addition, the frequent acquisition of drug-resistance is often associated with chemotherapy and is a significant obstacle to achieving favorable outcomes. Thus, exploring novel targets for therapy and developing more effective treatment strategies for this disease is required. Recently, Lauvrak *et al* (29) identified that *miR-155* overexpression in OS cell lines was associated with aggressive cancer phenotypes. In the present study, the aim was to evaluate whether *miR-15*5 is a sensitive target for therapy. The regulatory role of *miR-155* was determined in the proliferation, invasion and migration of OS cells. Subsequently, the *miR-155* inhibitor was evaluated for its inhibition on the OS cell proliferation and migration. The results demonstrated that the *miR-155* mimic significantly increased, whereas the *miR-155* inhibitor significantly reduced the proliferation and migration of OS MG-63 cells. Therefore, the study revealed *miR-155* as a possible therapeutic target for OS.

## Materials and methods

### Reagents and cell culture

The human OS cell line, MG-63, was obtained from the Cell Resource Center of the Chinese Academy of Medical Sciences (Beijing, China). MG-63 cells were cultured in Eagle’s Minimum Essential Medium (EMEM) (Invitrogen, Carlsbad, CA, USA), supplemented with 2 mM glutamine, 1% non-essential amino acids and 10% fetal bovine serum (FBS) (Invitrogen). The cells were incubated at 37°C with 5% CO_2_. The *miR-155* mimic (Qiagen, Valencia, CA, USA) or inhibitor (Qiagen) was used to elevate or reduce the *miR-155* level via lipofectamine 2000 (Invitrogen). *miR-Con* was used as a control.

### RNA extraction and reverse transcription quantitative polymerase chain reaction (RT-qPCR) miR-155 assay

The mirVana miRNA Isolation kit (Ambion, Austin, TX, USA) was used to extract miRNAs from the MG-63 cells, and the mirVana RT-qPCR miRNA Detection kit (Ambion) was used to quantify the *miR-155* expression, with the U6 small nuclear RNA as the internal control. ΔΔCt method was used for relative quantification (30). The RT-qPCR was performed using SYBR Green with the LightCycle 2.0 (Roche Diagnostics GmbH, Mannheim, Germany).

### Cell viability assay and cell colony formation assay

The MTT assay was adopted to determine the cell viability. MG-63 cells were seeded in 96-well plates and transfected with the *miR-155* mimic, inhibitor or control, with ~85% confluence. The cells were washed with warm PBS 6 h post-tranfection and were replaced with RPMI-1640 medium containing 1% FBS, and were cultured for various time. Subsequently, the MTT assay was conducted. Briefly, the incubation medium in the cell wells was replaced with 50 μl 1× MTT solution, and the cells were incubated for 2 h at 37°C. Post-incubation, the MTT solution was discarded and 150 μl DMSO was added to dissolve the precipitate completely at room temperature. The optical density was measured at 570 nm using a spectrophotometer, the cell viability was expressed as relative viable cells (%) to the control MG-63 cells. For the cell colony formation assay, 2×10^3^ cells were incubated in 6-well plates at 37°C containing 5% CO_2_. Ten days post-incubation, the cells were stained with crystal violet (0.005%) for 30 min and the colony numbers were recorded by Image J software (National Institutes of Health, Bethesda, MD, USA). For the proliferation assay, post-transfection with the *miR-155* mimic, inhibitor or control, cells were incubated in cell counting kit 8 (CCK-8; Dojindo Laboratories, Kumamoto, Japan) for various times. The 450 nm absorbance of each well was detected following visual color occurrence.

### Cell migration and invasion assay

The cell migration was determined by the scratch assay. The cells were cultivated to 90% confluence on 12-well plates and were transfected with the *miR-155* mimic, inhibitor or control. Subsequently, Cell Scrapers (Corning Inc., Corning, NY, USA) were utilized to scratch the confluent cells 24 h post-transfection. The procedures of cellular growth were observed at 0 and 96 h. All the experiments were repeated in triplicate. The Transwell migration chambers were used to evaluate the MG-63 cell invasion. The cells were first seeded at a density of 1×10^5^ cells in serum-free media on the upper chamber with the non-coated membrane (8 μm pore size; Millipore, Zug, Switzerland). The lower chamber contained EMEM with 20% FBS as a chemoattractant. The cells in the upper chamber were discarded using cotton wool after 24 h and the migration cells in the lower chamber were counted using a microscope (Olympus, Tokyo, Japan). All the experiments were repeated in triplicate.

### Statistical analysis

The results are expressed as mean ± standard error. Student’s t-test was performed to compare the differences between two groups. Statistical analysis was conducted by SPSS 17.0 software (SPSS, Inc., Chicago, IL, USA). P<0.05 was considered to indicate a statistically significant difference; and in particular, the results are shown as no significance, ^*^P<0.05, ^**^P<0.01 or ^***^P<0.001.

## Results

### miR-155 inhibitor reduces the viability and proliferation of MG-63 cells

**Figure 1:**
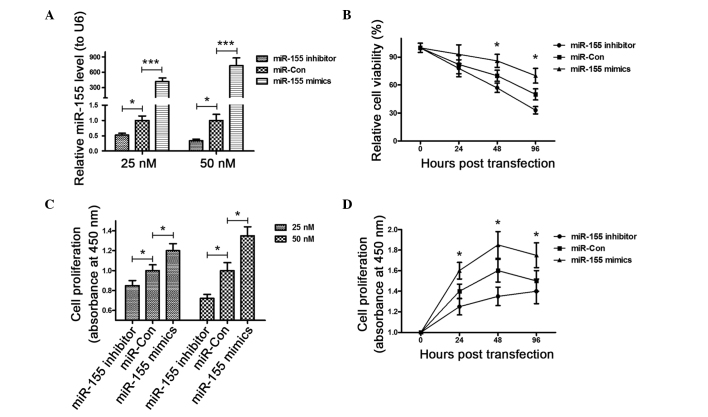
*miRNA-155* inhibitor reduces the cellular viability and proliferation of MG-63 cells *in vitro*. (A) The *miR-155* level in MG-63 cells was compared among the *miR-155* mimic, inhibitor and control transfection groups. (B) Viability of MG-63 cells was determined with the MTT assay post-transfection of the *miR-155* mimic, inhibitor and control. (C) Cellular proliferation of MG-63 cells post-*miR-155* mimic, inhibitor or control transfection at 25 nM or 50 nM by the CCK-8 assay. (D) Growth curve of cell proliferation was made following treatment with the *miR-155* mimic, inhibitor or control in MG-63 cells by the CCK-8 assay. All the experiments were performed separately in triplicate. ^*^P<0.05, ^**^P<0.01 and ^***^P<0.001. CCK-8, cell counting kit 8.

To confirm the promotion of *miR-155* to the OS cell proliferation, the *miR-155* expression level was manipulated in MG-63 cells, via transfection with the *miR-155* mimic or inhibitor. The *miR-155* in mimic-transfected cells was significantly higher than that of the control cells (P<0.001) 48 h post transfection, whereas the *miR-155* level in the *miR-155* inhibitor-transfected cells was significantly lower than in the control cells (P<0.05) (Fig. 1A). Subsequently, the influence of the *miR-155* mimic, inhibitor or control on the cell viability was examined. The MTT assay results (Fig. 1B) demonstrated that the viability of the MG-63 cells 48 h post-transfection decreased significantly following the transfection of the *miR-155* inhibitor compared to the transfection of *miR-Con* (P<0.05); whereas the transfection of the *miR-155* mimic ameliorated the viability reduction of MG-63 cells (P<0.05). Finally, the proliferation of MG-63 cells was determined post-transfection for 24 h with the *miR-155* mimic, inhibitor or control in a 25 or 50 nM concentration by the CCK-8 assay. Fig. 1C shows that in either concentration, the *miR-155* mimic group exhibited a higher proliferation than *miR-155* control, whereas the *miR-155* inhibitor group reduced proliferation (P<0.05). In addition, the time-dependent promoting or reducing effect in cell proliferation of the *miR-155* mimic or inhibitor was indicated under the condition of enhanced or reduced *miR-155* levels in the MG-63 cells (P<0.05) (Fig. 1D).

### miR-155 inhibitor reduces clone formation of MG-63 cells

**Figure 2:**
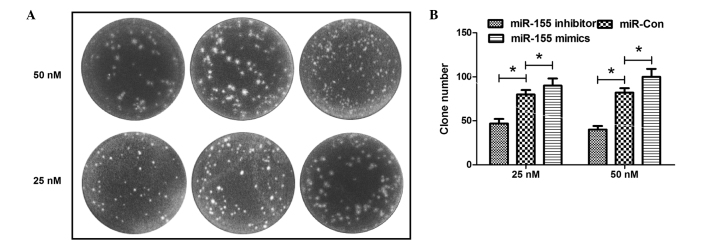
*miRNA-155* inhibitor reduces the colony formation of MG-63 cells. (A) MG-63 cells were transfected with the *miR-155* mimic, inhibitor or control at 25 nM or 50 nM, and were detected for colony formation. (B) The morphological characteristics of MG-63 colony formation and the number of colony formation was calculated as comparison. All the results were from experiments performed separately in triplicate. ^*^P<0.05.

The difference in colony formation was also detected for the MG-63 cells transfected with the *miR-155* mimic, inhibitor or control in the 25 or 50 nM concentration. The image of the colonies is shown in Fig. 2A, and the MG-63 cells that were transfected with the *miR-155* mimic in a 25 or 50 nM concentration formed more colonies than the *miR*-control-transfected cells, whereas the *miR-155* inhibitor reduced the colony formation of MG-63 cells (P<0.05) (Fig. 2B). All these findings indicate that the *miR-155* inhibitor reduced the clonegenesis of MG-63 cells, while the upregulated *miR-155* in the cells had a significant role in enhancing the proliferative capability and colony formation of the MG-63 cells.

### miR-155 inhibitor reduces the migration and invasion of MG-63 cells

**Figure 3:**
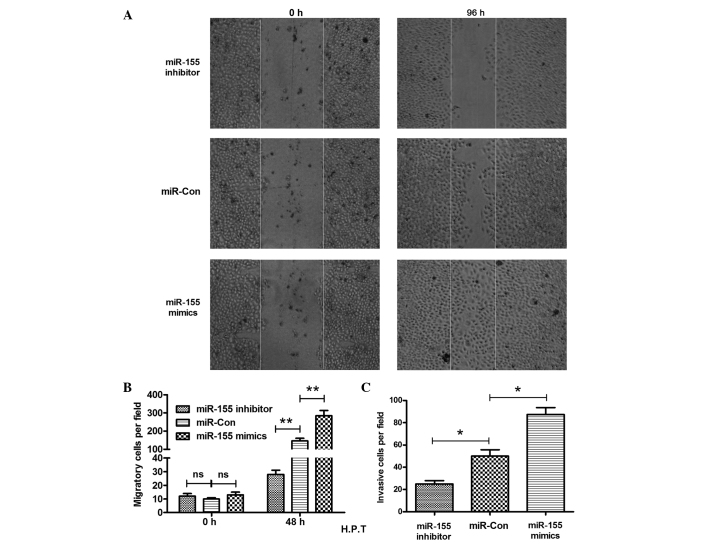
*miRNA-155* inhibitor reduces the migration and invasion of MG-63 cells. (A) Post-transfection with *miR-155* mimic, inhibitor or control, the MG-63 cells were shown at 0 or 96 h by the scratch assay. Solid lines are shown as a baseline. (B) The migratory cells were counted respectively in the *miR-155* mimic, inhibitor or control groups. (C) The number of tumor cell invasion was calculated to compare the *miR-155* mimic, inhibitor or control groups by the Transwell invasion assays. Experiments were performed separately in triplicate. ns, no significance; ^*^P<0.05; ^**^P<0.01.

Cell migration is known to contribute to tumor metastasis (31). The migration of the MG-63 cells was determined post-transfection of the *miR-155* mimic, inhibitor or control by the scratch assay. The results shown in Fig. 3A indicate that more inoculation occurred 96 h post-scratch. The MG-63 cells post *miR-155* mimic-transfection migrated significantly faster than the *miR-Con*-transfected MG-63 cells, as there were more cells crossing the base line (P<0.01) (Fig. 3B). In addition, the *miR-155* inhibitor reduced the migration of MG-63 cells significantly, as less cells crossed the base line in this group than in the control group (P<0.01) (Fig. 3B). The *miR-155* inhibitor clearly reduced the MG-63 cell migration. The blockage of the *miR-155* inhibitor to the cell invasion was also demonstrated. The Transwell invasion chamber assay demonstrated clearly that there was a significant difference in the cell invasion between the *miR-155* mimic and control groups, or between the *miR-155* inhibitor and control groups. The number of invasive cells was 50±10 cells in the control group, whereas the invasive cell number in the *miR-155* mimic or inhibitor group was 88±12 and 25±4 cells, respectively (Fig. 3C) (P<0.05, respectively). All the results indicated that overexpression of *miR-155* stimulated the migration and invasion of OS cells, and the *miR-155* inhibitor reduced the migration and invasion of the MG-63 cells.

## Discussion

As the most common malignant primary bone tumor in childhood (32), OS maintains a high recurrence of 30–40%, and 80% of OS patients with metastatic disease at diagnosis will relapse (27,33,34), regardless of the significant improvements in the overall survival rate of high-grade OS patients during the past decades. Failure of standard multimodal therapy for the disease is associated with an extremely poor prognosis, and therefore, novel drugs or combination therapies are required for patients with recurrent or refractory high-grade OS. Several clinical studies have been conducted to evaluate the efficiency of a combined therapy with gemcitabine and docetaxel in recurrent or refractory OS, and the effect of the gemcitabine-docetaxel combination regimen in recurrent or refractory OS patients remains controversial (35–37).

Extensive studies have been conducted to identify the oncogenes that are suitable to become targets of monoclonal antibodies and small inhibitors. Antibodies or inhibitors were used to knockdown the tyrosine kinase receptors, KIT, platelet-derived growth factor receptors and vascular endothelial growth factor receptors (38–41), however, their inhibition lacked antitumor activity. The monoclonal antibody anti-insulin-like growth factor receptor-I was also promising preclinically, but was not confirmed to be effective in the clinical setting (42). Recently, several studies have focused on the signal transduction pathways of phosphatidylinositol 3′-kinase/mammalian target of rapamycin (43) and mitogen-activated protein kinases. Their inhibition proved highly effective in OS preclinical models (44).

Previously, various miRNAs have been confirmed to be deregulated in OS (14,15). Several oncogenic miRNAs, including *miR-21*, *miR-93* and *miR-29,* have been indicated to be overexpressed and to induce cancer cell growth, migration, invasion and metastasis (16–19,45). Recently, the *miR-155* dysregulation in OS was discovered by microarray analysis (29). In the present study, the regulation of *miR-155* was explored on the OS cell proliferation, migration and invasion on the MG-63 cell *in vitro*. The *miR-155* mimic was shown to promote the cell proliferation, colony formation, migration and invasion significantly, compared to the control miRNA. An *miR-155* inhibitor was also used to evaluate whether *miR-155* could serve as a therapeutic target for OS. The results demonstrated that the *miR-155* inhibitor significantly reduced the proliferation, colony formation, migration and invasion of MG-63 OS cells.

In conclusion, the present study confirmed that the oncogenic regulation on the OS progression of *miR-155* could serve as a therapeutic target with an *miR-155* inhibitor.
